# The Role of Kinase Modulators in Cellular Senescence for Use in Cancer Treatment

**DOI:** 10.3390/molecules22091411

**Published:** 2017-08-25

**Authors:** Chang Sup Lee, Juhwa Baek, Sun-Young Han

**Affiliations:** College of Pharmacy and Research Institute of Pharmaceutical Sciences, Gyeongsang National University, 501 Jinju-daero, Jinju, Gyeongnam 52828, Korea; changsup@gnu.ac.kr (C.S.L.); qorwnghk2006@naver.com (J.B.)

**Keywords:** aging, cancer, kinase inhibitor, senescence

## Abstract

Recently, more than 30 small molecules and eight monoclonal antibodies that modulate kinase signaling have been approved for the treatment of several pathological conditions, including cancer, idiopathic pulmonary fibrosis, and rheumatoid arthritis. Among them, kinase modulators have been a primary focus for use in cancer treatment. Cellular senescence is believed to protect cells from tumorigenesis by irreversibly halting cell cycle progression and avoiding the growth of damaged cells and tissues. Senescence can also contribute to tumor suppression and be utilized as a mechanism by anti-cancer agents. Although the role of kinase modulators in cancer treatment and their effects on senescence in tumor development have been extensively studied, the relationship between kinase modulators for cancer treatment and senescence has not been fully discussed. In this review, we discuss the pro- and anti-tumorigenesis functions of senescence and summarize the key roles of kinase modulators in the regulation of senescence against tumors.

## 1. Introduction

**Figure 1:**
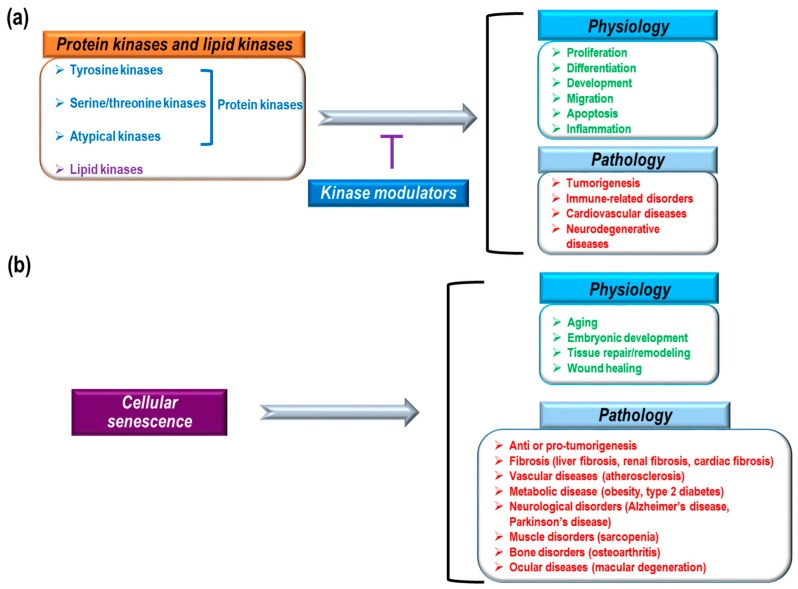
Physiological and pathological roles of protein and lipid kinases and cellular senescence. (**a**) 518 kinases (protein kinases (blue): tyrosine kinases, serine/threonine kinases, and atypical kinases; lipid kinases (purple)); and (**b**) cellular senescence has a variety of physiological (green) and pathological (red) functions.

Previous research, including human genome-wide studies, have identified and characterized a variety of human protein and lipid kinases [1,2,3,4]. The identified protein kinases consist of 478 eukaryotic protein kinases (388 serine/threonine and 90 tyrosine kinases) and 40 atypical kinases [2,5]. These kinases, as core enzymes mediating intra- and intercellular signaling, are highly implicated in a variety of physiological and pathological conditions such as proliferation, differentiation, development, migration, apoptosis, inflammation, tumorigenesis, immune-related disorders, and cardiovascular diseases (Figure 1a) [6,7,8,9,10,11,12,13]. Therefore, many pharmacological studies have focused on kinase inhibitors to treat severe pathological conditions. To date, the U.S. Food and Drug administration (FDA) has approved about 30 small molecule inhibitors and eight monoclonal antibodies that regulate kinases to treat cancer, idiopathic pulmonary fibrosis, and rheumatoid arthritis [14]. Small molecule kinase inhibitors can be classified into seven groups based on their manner of binding to kinases [15,16,17,18,19]. Type I inhibitors can compete with ATP-binding sites in the active states of kinases. Type I½ inhibitors and Type II inhibitors can interact with ATP-binding pockets in the inactive DFG (Asp-Phe-Gly)-Asp in conformation and inactive DFG-Asp out conformation of kinases, respectively. Type III inhibitors have binding sites in allosteric pockets to block ATP loading into kinases but do not compete with ATP. Type IV inhibitors have allosteric binding sites that are distant from ATP-binding pockets. Type V inhibitors interact with two different parts of kinases and Type VI inhibitors interact covalently with kinases. Among these groups of kinases, most approved drugs are type I and II inhibitors [17]. In addition, most drugs inhibit tyrosine kinases, whereas relatively few inhibit serine/threonine kinases (B-RAF inhibitor: vemurafenib, MEK1/2 inhibitor: trametinib, cyclin dependent kinase [CDK] inhibitor: palbociclib) and even fewer inhibit lipid kinases (PI3Kδ inhibitor: idelalisib) [17,20,21,22,23,24]. Currently FDA-approved monoclonal antibodies target EGFR, HER2, and VEGF signaling. These antibodies exert anti-cancer effects by blocking kinase signaling and/or antibody-dependent cytotoxicity. Small molecules and antibodies have different ways of inhibiting kinases. Small molecules can penetrate cell membranes and work inside of cells. Also, small molecules often compete with ATP for binding in the ATP-binding pocket of kinases, or they induce conformational changes in kinases to block their activity [15,16,17,18]. Although small molecule inhibitors regulate kinase activity via direct interactions, it has also been reported that many small molecules have non-kinase targets and various off-target binding sites with irrelevant kinases [18,25]. Therefore, in addition to direct regulation of kinase activity by small molecules, there could be indirect inhibition, such as modulation of interactions between kinases and other proteins, as well as off-target effects. However, antibodies cannot pass through cell membranes to enter cells. Antibodies targeting tyrosine kinase receptors inhibit kinase signaling through several mechanisms such as blocking ligand binding sites, obstructing receptor dimerization by steric hindrance, and inhibiting downstream signaling through interactions with the juxtamembrane domain of receptors [26,27]. Furthermore, antibodies can mediate complement-dependent cytotoxicity, complement-dependent cell-mediated cytotoxicity, and antibody-dependent cytotoxicity by interacting with complement factors and immune effector cells (macrophages and natural killer cells) [26,27]. Although antibodies have been known to be more target-specific than small molecules, antibodies also have adverse effects due to weak tissue specificity and hypersensitivity reactions [28,29].

Cellular senescence is the irreversible arrest of cell proliferation, which prevents aberrant cells from multiplying and dividing [30,31,32,33,34]. Blocking further proliferation of damaged cells is a key mechanism for stopping tumorigenesis [30,31,32,33]. Therefore, it is believed that cellular senescence mediates anti-tumor processes [35,36,37]. In addition to blocking tumor development, cellular senescence has also been implicated in many physiological processes, including aging, embryonic development, tissue repair/remodeling, and wound healing [32,38,39,40,41,42,43]. Cellular senescence is also thought to play a role in pathological processes, including liver fibrosis [44], renal fibrosis [45], cardiac fibrosis [46], vascular diseases (atherosclerosis) [47,48], metabolic diseases (obesity [49], type 2 diabetes [50]), neurological disorders (Alzheimer’s disease [51], Parkinson’s disease [52]), muscle disorders (sarcopenia) [53], bone disorders (osteoarthritis) [54], and ocular diseases (macular degeneration [55]) (Figure 1b). Based on the mechanism, cellular senescence can be categorized as replicative senescence and stress-induced premature senescence [33]. Replicative senescence is closely linked to telomere shortening in a cell proliferation-dependent manner [56,57]. Continuous cell proliferation results in telomere shortening, the length of which represents the number of cell divisions [58]. Telomere shortening to a critical point induces a DNA damage response (DDR) that causes the arrest of cell division and mediates cellular senescence [59]. The DDR-activated P53 pathway can increase P21 expression levels to transduce senescence signaling [33,60]. DNA damage-induced senescence is caused by genotoxic damage from UV and ionizing radiation as well as oxidative stress [61,62]. Stress-induced senescence is mainly caused by an increase in reactive oxygen species (ROS) that can activate the P38 MAPK pathway, leading to the activation of P53 and P21 [63,64]. Oncogene-induced senescence begins with overexpression of a RAS oncogene in primary cells [37]. Currently, about 50 oncogenes are known to induce senescence, which can be mediated by the P16, DDR-P53-P21, and ARF-P53-P21 pathways [65,66,67,68]. Oncogene-induced senescence provides strong evidence that the senescence process has an anti-tumorigenic effect during early cancer development [69]. Along with a gain of function in oncogenes (RAS, MYC, AKT, Cyclin E, etc.), loss of function of tumor suppressor genes (PTEN, NF1, and VHL) can also induce cellular senescence [31,33]. P53, a well-known tumor suppressor that functions against tumor development, is also a key mediator of cellular senescence [70,71]. P53 can integrate many cellular stress signals, including DNA damage, nutrient deprivation, oncogene activation, and hypoxia [71,72]. In response to genotoxic damage, phosphorylation of P53 (serine 15 residue) by ataxia telangiectasia mutated kinase (ATM) and ATM-related kinase (ATR) mediates the activation and stabilization of P53 [70,71]. Most FDA-approved kinase inhibitors are used for cancer treatment [14]. Because kinase signaling is critically involved in tumorigenic processes, such as cell survival, cell motility, immune evasion, cell proliferation, DNA damage response, angiogenesis, and metabolism [73], much pharmacological and therapeutic research on the development of kinase inhibitors converges on tumor biology and cancer treatment. Cellular senescence, as one potential cellular fate, is also associated with a variety of kinase signaling pathways [33]. Here, we discuss the roles of cellular senescence and aging in the process of cancer development; we also summarize which kinase inhibitors used for cancer treatment affect senescence processes.

## 2. Cancer and Senescence

Tumor cells are characterized and defined by uncontrolled growth and unlimited proliferation. Tumorigenesis is caused by the integration of multiple processes, such as DNA instability, stromal microenvironmental changes, aberrant proliferation, and immune system alternations [74]. Cellular senescence is characterized by irreversible cell arrest. Tumor cells display the opposite of cellular senescence, which has the tract of irreversible cell arrest [31,33], however, tumor development is also closely linked to various cellular senescence phenotypes [31,33].

**Figure 2:**
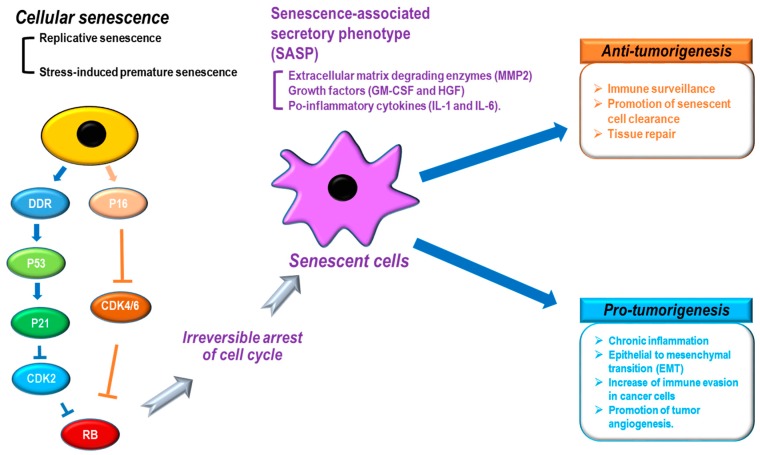
Senescence-associated secretory phenotype (SASP) and tumorigenesis. Cellular senescence by multiple causes: (black) shows distinct phenotypes, including SASP (purple) that can induce anti-tumor (orange) or pro-tumor (sky-blue) development. DDR: DNA damage response, CDK: cyclin-dependent kinase, MMP2: matrix metalloproteinase 2, GM-CSF: granulocyte-macrophage colony-stimulating factor, HGF: hepatocyte growth factor.

When cellular senescence is induced, cells show distinct characteristics, such as morphological changes (cell and nuclear size enlargement, cell flattening, vacuolization, and stress granules), protein alternations (overexpression of senescence-associated β-galactosidase [SA-β-gal] and P16), chromatin remodeling (senescence-associated heterochromatin foci [SAHF] formation and lamin B1 loss),and the senescence-associated secretory phenotype (SASP: secretion of extracellular matrix degrading enzymes [MMP2], growth factors [GM-CSF and HGF], and pro-inflammatory cytokines [IL-1 and IL-6]) (Figure 2) [31,34]. In particular, the SASP shows a variety of cellular effects on neighboring cells [31]. For tumor development, the SASP is known to function as both a tumor promoter and suppressor [31,75,76,77]. The SASP can negatively contribute to tumorigenesis by increasing immune surveillance, promoting senescent cell clearance, and repairing tissues (Figure 2). In contrast, the SASP can also potentiate cancer development through tissue dysfunction via chronic inflammation, epithelial to mesenchymal transition (EMT), increasing immune evasion in cancer cells, and promoting tumor angiogenesis (Figure 2). In addition, although senescence is implicated in removing damaged cells and tissue to protect against cancer development, senescence has also been identified as a key contributor to aging [78,79] and aging-related diseases [80]. Thus, the question arises whether the role of senescence is to mediate tumor suppression or tumor development? Acute/transient senescence to eliminate damaged cells can give rise to beneficial effects such as tumor suppression, whereas chronic/long-term senescence can result in the accumulation of senescent cells with detrimental effects such as tumor promotion [31,33]. During aging, an increase in the number of senescent cells can be accompanied by dysfunction of the SASP, the DNA repair system, and immune function [31]. The eventual integration of these multiple senescence processes can cause premalignant cells to develop into cancerous cells.

These close relationships between senescence and tumor development have led researchers to investigate chemical inhibitors that modulate senescence and cancer. Senescence is reportedly regulated by multiple types of chemical inhibitors, including DNA topoisomerase inhibitors (etoposide, doxorubicin, and daunorubicin), DNA cross linkers (cyclophosphamide, mitomycin C, and cisplatin), and cyclin-dependent kinase inhibitors (ribociclib, roscovitine, and palbociclib) [30]. Most of the tested compounds have been shown to block the proliferation of cancer cell lines in previous studies [30]. Although there are many studies on the effects of various chemical inhibitors on cancer development and senescence, in this study we focus on the role of FDA-approved kinase modulators in the senescence process for cancer treatment.

## 3. Kinase Modulators and Senescence

### 3.1. BCR-ABL Inhibitors: Imatinib and Dasatinib

Imatinib and dasatinib are BCR-ABL kinase inhibitors approved for the treatment of chronic myeloid leukemia (CML). A shortened chromosome 22 is observed in CML patients, which has been designated the Philadelphia chromosome. The Philadelphia chromosome is the product of a translocation between chromosomes 9 and 22, resulting in the fusion of the *bcr* and *abl* genes and the generation of fusion protein BCR-ABL. Small molecules inhibiting the kinase activity of the ABL protein were developed and exhibited anti-leukemic activity, leading to dramatic therapeutic effects in CML [81].

Imatinib, also known as STI-571, Gleevec^®^ and Glivec^®^, was the first kinase inhibitor developed for clinical use and began the era of molecularly targeted therapy. BCR-ABL is one of the main targets of imatinib, and apoptosis is known to be its primary mechanism for growth suppression. Other mechanisms of growth inhibition by imatinib are also being explored such as senescence and autophagy. Treatment of the K562 CML cell line with imatinib has resulted in cellular senescence, as indicated by an increase in SA-β-gal positive cells and the induction of cell cycle inhibitor P27 [82]. It was also shown that imatinib-induced senescence was closely associated with autophagy and apoptosis. Blocking apoptosis potentiated senescence, whereas autophagy inhibited the senescence response.

Dasatinib (Sprycel^®^) is a second-generation BCR-ABL inhibitor with improved potency and better inhibitory profiles against the ABL mutants found in CML patients [83]. Sen et al. reported the induction of senescence by dasatinib [84]. Although dasatinib is a well-known BCR-ABL inhibitor, it was used in that study as a multitargeted kinase inhibitor of non-small cell lung cancer (NSCLC) with the EGFR-activating mutation. During the clinical study of dasatinib for NSCLC patients, one patient showed a dramatic response and continued to improve long after dasatinib treatment was stopped. That patient was found to have a kinase inactive B-RAF mutation. Cell lines expressing this B-RAF mutation were generated, and treatment with dasatinib resulted in senescence and apoptosis. Senescence induction was confirmed by cell cycle arrest, decreased proliferation, SA-β-gal staining, and heterochromatin protein 1-γ (HP1-γ) staining [82]. Thus, senescence induced by dasatinib was found to contribute to its therapeutic effect on NSCLC with kinase-inactivating BRAF mutations. Another study exploring the mechanism of dasatinib-induced senescence has since been reported [85]. These results suggest that dasatinib induced DNA damage and the DNA repair pathway, leading to cellular senescence.

### 3.2. EGFR Family Modulators: Gefitinib, Erlotinib, Lapatinib, and Cetuximab

Inspired by the success of imatinib as an anti-leukemic agent, other drugs and antibodies have been developed targeting other kinases. The epidermal growth factor receptor (EGFR) signaling pathway was the obvious next target due to its deep association with tumor development in various cancer types, including lung and colorectal cancer [86]. Cetuximab and panitumumab are monoclonal antibodies against EGFR, and gefitinib, erlotinib, afatinib, and osimertinib are small molecule inhibitors of EGFR kinase activity. Lapatinib is a small molecule dual kinase inhibitor against both EGFR and human epidermal growth factor receptor 2 (HER2, also known as ERBB2 or NEU) [86].

Gefitinib (ZD1839, Iressa^®^), the first EGFR tyrosine kinase inhibitor approved by the FDA, is indicated for the treatment of a subset of NSCLC. Reports by Hotta et al. elucidated senescence induction in NSCLC cells as a mechanism of gefitinib’s antitumor activity [87]. Gefitinib treatment of NSCLC cell lines increased the number of senescent cells, as indicated by enlarged and flattened morphology, SA-β-gal staining, and upregulation of CDK inhibitors (P16, P27, and P21). Additionally, ex vivo exposure of NSCLC tumor cells to gefitinib also induced senescence.

Erlotinib (Tarceva^®^) is another EGFR small molecule inhibitor approved by the FDA for the treatment of NSCLC with specific EGFR mutations and pancreatic cancer [83]. Erlotinib’s role in cellular senescence was also reported in two studies [88,89]. One study reported its effects on cervical cancer cells known to be related to human papilloma virus (HPV) infection. Oncogenes E6 and E7 are encoded by HPV type 16 and expressed in cervical carcinomas; E6 and E7 cause immortalization of cervical epithelial cells, leading to cervical carcinogenesis. EGFR inhibition by erlotinib was shown to prevent immortalization by inducing senescence and apoptosis in cervical cancer cells [88]. Erlotinib treatment also increased the population of SA-β-gal positive cells in a subpopulation of E6/E7-infected cells. The second study showed that treatment with EGF suppressed cellular senescence in human epithelial cells, whereas removal of EGF from culture media induced cellular senescence. Cellular senescence was measured by enlarged morphology, elevated SA-β-gal activity, reduced retinoblastoma (Rb) protein phosphorylation, and increased P21 expression. Consistent with these results, erlotinib treatment also induced senescence and premature aging in vivo in mice that were administered erlotinib [89].

Lapatinib (Tykerb^®^), an EGFR/HER2 dual inhibitor, is clinically used for patients with HER2 positive breast cancer, and the senescence-related effect of lapatinib has been explored in breast cancer cells. Unlike other kinase inhibitors, there are reports on lapatinib’s effects on both senescence induction and suppression. One study reported the senescence suppressing effect of lapatinib [90]. Overexpression of HER2 by breast cancer cells resulted in cellular senescence, which was dose-dependently blocked by lapatinib treatment. SA-β-gal staining, immunostaining of P21, γH2AX, 53BP1, SASP (IL-6, IL-8, and AREG), and morphological changes were employed as markers of senescence. Oncogene-induced senescence (OIS) has a paradoxical effect on tumorigenesis. While OIS is considered to be a tumor suppression mechanism [91], pro-tumorigenic molecules are also secreted by senescent cells, manifesting the senescence-associated secretory phenotype (SASP) [92]. OIS may be pro-tumorigenic or anti-tumorigenic, depending on the context. In one study, EGF induced senescence, which was blocked by lapatinib treatment [90].

Other studies have supported the role of lapatinib in senescence induction. Lapatinib was evaluated in one study as a radiosensitizer in breast cancer cells [93]. Lapatinib potentiated radiation-induced cell death and induced cellular senescence as indicated by the increased percentage of SA-β-gal positive cells. Meeting abstracts by McDermott et al. also reported the role of lapatinib as a senescence inducer. Long-term, low-dose treatment of lapatinib resulted in a senescent-like phenotype as indicated by altered morphology and SA-β-gal staining [94,95]. Due to the conflicting results of previous reports, further research is required to elucidate lapatinib’s role in senescence.

Cetuximab (Erbitux^®^) is an FDA-approved monoclonal antibody used to treat colorectal and head-and-neck cancer. By binding to EGFR, cetuximab blocks phosphorylation and activation of EGFR, thereby inhibiting the signaling pathway. Thus, cetuximab induces growth inhibition and apoptosis in cells requiring EGFR signaling [96]. In order to expand the indication of cetuximab, cetuximab was also investigated in gastric cancer cell lines [97]. Cetuximab treatment alone did not affect cell growth in gastric cancer cells; however, cetuximab sensitized the effect of the chemotherapeutic agent irinotecan when used in combination therapy. While cetuximab enhanced both the apoptosis and cell cycle arrest induced by irinotecan, SA-β-gal activity was unchanged by the combination treatment. Therefore, senescence in gastric cancer cell lines was not affected by cetuximab treatment either alone or in combination with irinotecan. 

### 3.3. Angiogenesis Inhibitors: Sunitinib, Axitinib, and Bevacizumab

A key feature of cancer development is angiogenesis, wherein new capillaries are formed to provide nutrients and oxygen to tumor cells. Angiogenesis is primarily mediated by vascular endothelial growth factor (VEGF) signaling in vascular endothelial cells. Stimulation of the VEGF receptor (VEGFR) by VEGF results in cell proliferation, migration, and invasion [98]. Blocking the tumor’s vascular supply has been a target of cancer therapy, and the development of anti-angiogenesis therapy has been intensively investigated. Bevacizumab, a monoclonal antibody against VEGF, was the first approved anti-angiogenesis therapy. Small molecule inhibitors of VEGFR’s kinase activity have also been developed for anti-angiogenesis. Small molecule drugs containing VEGFR inhibitory activity include sunitinib, sorafenib, pazopanib, vandetanib, regorafenib, axitinib, and lenvatinib. These drugs are also called multikinase inhibitors, because they affect multiple kinases other than VEGFR, which contributes to their anti-cancer effects. Beyond inhibiting angiogenesis, these drugs exert anti-proliferative effects on cancer cells, directly leading to therapeutic results. Senescence is one anti-tumor mechanism utilized by these multikinase inhibitors. Senescence-related effects were reported for sunitinib, axitinib, and bevacizumab.

Sunitinib (SU11248, Sutent^®^) is a small molecule drug that targets multiple kinases and is approved for the treatment of renal cell carcinoma (RCC) and gastrointestinal stromal tumor (GIST). Sunitinib’s senescence-inducing effect was elucidated in metastatic RCC [99]. Treatment of RCC cell lines with sunitinib induced senescence characteristics such as SA-β-gal activity, the SASP (IL-1, IL-6, and IL-8), and expression of Decoy Receptor 2 (DcR2) and Deleted in Esophageal Cancer 1 (Dec1).

Axitinib (AG013736, Inlyta^®^), a potent inhibitor of VEGFR1, 2, and 3, was approved in 2012 for the treatment of RCC. The direct cytotoxic mechanism of axitinib in RCC was investigated by Morelli [100]; axitinib was found to induce the DNA damage response leading to senescence with an increased level of SA-β-gal activity. Senescence induced by axitinib was dependent upon reactive oxygen species generation, as indicated by reduced SA-β-gal activity after pre-treatment with *N*-acetyl cysteine [100]. The same group reported the effect of axitinib on senescence in glioma cell lines [101]. In a cytotoxicity assay, U87 and T98 glioma cell lines were sensitive to axitinib treatment; in contrast, resistance to cytotoxicity by axitinib was observed in U251 glioma cells. Consistent with these varied results, senescence was induced by axitinib in U87 and T98 cells, but not in U251 cells, as indicated by SA-β-gal activity.

Bevacizumab (Avastin^®^), which blocks angiogenesis by binding to VEGF and inhibiting its interaction with VEGFR1/2 receptors, is approved for the treatment of several cancer types, including metastatic colorectal cancer (CRC), NSCLC, and RCC [96]. Senescence induction by bevacizumab in colorectal cancer was investigated by Hasan et al. [102]. Cell viability was not affected by bevacizumab treatment in vitro in CRC cell lines. However, senescent growth arrest was caused by bevacizumab in both in vitro and in vivo tumor xenografts, as measured by increased SA-β-gal activity. Expression of P16 was associated with bevacizumab-induced senescence, demonstrating the role of P16 in senescence. Importantly, cellular senescence was also observed in tumor samples from patients who received bevacizumab treatment.

### 3.4. B-RAF Inhibitor: Vemurafenib

Vemurafenib (PLX4032, Zelboraf^®^) is an inhibitor of the mutant B-RAF gene, which has a V600E mutation that results in constitutive activation of B-RAF. This B-RAF mutation is found in the majority of cutaneous melanoma patients, and treatment with vemurafenib resulted in significant clinical improvement [103].

Induction of senescence by vemurafenib was reported in melanoma cells in vitro and in vivo [104]. In that report, the therapeutic effect of vemurafenib was shown to be mediated by apoptosis, cell cycle arrest, and senescence in both in vitro and in vivo xenograft studies. Senescence induced by vemurafenib was indicated by elevated SA-β-gal activity, enlarged cell morphology, and increases in H3K9me3 and promyelocytic leukemia (PML) protein as markers of nuclear heterochromatin accumulation.

### 3.5. MEK Inhibitor: Trametinib

Trametinib (GSK-1120212, Mekinist^®^) is a selective allosteric inhibitor of MEK1/2, which is a component of the MAPK signaling pathway [105]. The MAPK signaling pathway is constitutively activated by the B-RAF V600E/K mutation; thus, MEK1/2 is a good therapeutic target for melanoma harboring a B-RAF mutation [106]. Currently, the only approved indication for trametinib is B-RAF V600E/K mutation-positive metastatic melanoma.

The effect of trametinib on senescence was observed in combination with radiotherapy for melanoma treatment [107]. Treatment of trametinib enhanced cytotoxicity with radiotherapy, and this radiosensitization was mediated by senescence induction and cell cycle arrest. Trametinib pretreatment increased the percentage of senescent cells with high SA-β-gal activity in irradiated melanoma. Thus, senescence also contributed to the anti-tumor effect of trametinib.

### 3.6. CDK Inhibitor: Palbociclib

Palbociclib (PD-0332991, Ibrance^®^) is a CDK 4/6 inhibitor used to treat metastatic breast cancer. CDKs regulate the cell cycle machinery and act in association with regulatory subunits called cyclins. CDK4/6 form complexes with cyclin D, and control the G_1_ checkpoint [108]. Given the importance of CDKs in the cell cycle and proliferation, they have become an attractive target for cancer treatment. After 20 years of attempting to develop a CDK inhibitor, palbociclib was approved by the FDA for the treatment of advanced breast cancer in combination with letrozole.

In the study that investigated the mechanism of palbociclib’s effect on neutropenia, palbociclib did not cause senescence induction in the bone marrow mononuclear cells related to hematologic toxicity. However, MCF-7 breast cancer cell became senescent with palbociclib treatment, indicating a different mechanism of cytotoxic action between bone marrow and breast cancer cells [109]. Quantitative flow cytometry for SA-β-gal stained cells was used to assay cellular senescence.

## 4. Conclusions and Perspectives

In this study, we discussed FDA-approved kinase modulators that affect cellular senescence (Table 1). In addition to the direct killing of cancer cells, the main anti-tumor function of most kinase modulators is cell cycle arrest through the induction of cellular senescence.

However, unlike other kinase inhibitors, lapatinib (EGFR/HER2 inhibitor) is reported to inhibit cellular senescence, because the senescence marker (SA-β-gal) decreased in a dose-dependent manner [90]. Furthermore, palbociclib (CDK4/6 inhibitor) can induce cellular senescence in MCF-7 breast cancer cells but not in bone marrow mononuclear cells [109]. There are several explanations of these phenomena. First, kinase modulators may show different effects and roles in various cell types based on the cellular context [18]. In response to kinase modulators, some cells may be more susceptible to cellular senescence than others. Second, although many senescence markers, including SA-β-gal/P16 induction and the SASP, are used to indicate cellular senescence, these markers do not indicate that all cells become “truly senescent” in terms of absolute irreversible arrest of the cell cycle [110,111]. Although cells show a variety of expression levels of senescence markers, some cell types can restart the cell cycle under certain conditions [110,111]; thus, further research is needed to reveal how cellular senescence can vary by cell type and context. Third, although kinase inhibitors are well characterized by unique properties, such as their specific targets, cellular mechanisms, and in vivo side-effects, it is not clear how their off-target effects inhibit unknown targets. Many small molecule kinase inhibitors can share binding sites in the ATP-binding pockets of kinases [17,18]. The lack of target selectivity of kinase inhibitors can cause unwanted side effects, unpredicted results, and unknown phenomena in both cells and animal models.

Many factors still need to be investigated to understand the relationship between senescence, aging, and aging-related diseases. Although the main role of senescence is thought to be tumor repression, detailed studies are needed to characterize the exact role of senescence in cancer and aging as well as the acute and chronic effects of senescence. Moreover, while kinase inhibitors with multiple target selectivity might be a better choice for cancer treatment, reducing off-target effects is a challenging issue in clinical applications and basic research. Finally, our expanding knowledge of kinase modulators and senescence will contribute to the development of improved therapeutic methods in senescence- and aging-dependent diseases, including cancer.

## Figures and Tables

**Table 1:** FDA-approved kinase modulators and cellular senescence.

Drugs	Target	Effect on Senescence	References
Imatinib	BCR-ABL	Induction	[82]
Dasatinib	BCR-ABL	Induction	[84,85]
Gefitinib	EGFR	Induction	[87]
Erlotinib	EGFR	Induction	[88,89]
Lapatinib	EGFR, HER2	Suppression, Induction	[90,93,94,95]
Cetuximab	EGFR	No change	[97]
Sunitinib	VEGFR, multikinases	Induction	[99]
Axitinib	VEGFR, multikinases	Induction	[100,101]
Bevacizumab	VEGF	Induction	[102]
Vemurafenib	B-RAF	Induction	[104]
Trametinib	MEK1/2	Induction	[107]
Palbociclib	CDK4/6	Induction	[109]

## Abbreviations

The U.S. Food and Drug administration (FDA), Adenosine triphosphate (ATP), Cyclin dependent kinase (CDK), Phosphatidylinositol-4,5-bisphosphate 3-kinase δ (PI3Kδ), Human epidermal growth factor receptor 2 (HER2), DNA damage response (DDR), Oncogene-induced senescence (OIS), Ultraviolet (UV), Reactive oxygen species (ROS), Ataxia telangiectasia mutated kinase (ATM), ATM-related kinase (ATR), Mitogen-activated protein kinase (MAPK), Senescence-associated β-galactosidase (SA-β-gal), Senescence-associated heterochromatin foci (SAHF), Senescence-associated secretory phenotype (SASP), Matrix metalloproteinase 2 (MMP2), Granulocyte macrophage colony-stimulating factor (GM-CSF), Hepatocyte growth factor (HGF), Interleukin (IL), Epithelial to mesenchymal transition (EMT), Heterochromatin protein 1-γ (HP1-γ), Chronic myeloid leukemia (CML), Non-small cell lung cancer (NSCLC), Epidermal growth factor receptor (EGFR), Vascular endothelial growth factor receptor (VEGF), Human papilloma virus (HPV), Retinoblastoma (Rb), Renal cell carcinoma (RCC), Decoy receptor 2 (DcR2), Deleted in esophageal cancer 1 (Dec1), Metastatic colorectal cancer (CRC), Promyelocytic leukemia (PML).
